# Parasites and the ecology of fear: Nonconsumptive effects of ectoparasites on larvae reduce growth in simulated *Drosophila* populations

**DOI:** 10.1002/ece3.70079

**Published:** 2024-08-13

**Authors:** Collin J. Horn, Lien T. Luong, Darcy R. Visscher

**Affiliations:** ^1^ Department of Psychology and Neuroscience Dalhousie University Halifax Nova Scotia Canada; ^2^ Department of Biological Sciences University of Alberta Edmonton Alberta Canada; ^3^ Department of Biology The King's University Edmonton Alberta Canada; ^4^ Naturalis Biodiversity Center Leiden Netherlands

**Keywords:** computer simulation, Drosophila, ecology of fear, host‐parasite interactions, host population, Macrocheles, trait mediated effect

## Abstract

Predators negatively affect prey outside of direct attack, and these nonconsumptive effects (NCEs) may cause over half the impacts of predators on prey populations. This “ecology of fear” framework has been extended to host–parasite interactions. The NCEs of parasites are thought to be small relative to those of predators. However, recent research shows ectoparasites exert NCEs on multiple life stages of *Drosophila*. In this study, we apply recent data to a matrix‐based model of fly populations experiencing infection/consumption and NCEs from an ectoparasitic mite. We found the NCEs of parasites on larvae, which are not actively parasitized, decreased the size of simulated host populations. By contrast, the NCEs on adult flies increased population size through compensatory egg production. The negative NCEs on larvae outweighed the positive effects on adults to reduce population size. This study suggests that parasitic NCEs can suppress host populations independent of infection.

## Graphical abstract

Using simulations we tested if the nonconsumptive effects on different life stages of flies from ectoparasites affected population growth. Nonconsumptive effects on larvae and adults had conflicting outcomes, NCEs on larvae reduced population growth, and NCEs on adults slightly increased growth. When the NCEs were combined, the negative effect on larvae overpowered the positive effect on adults and reduced population size.

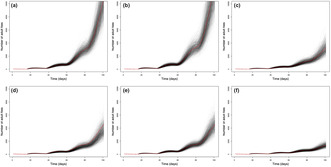

## 1 INTRODUCTION

The “ecology of fear” framework was developed to describe the negative effects of predators on their potential prey outside of consumption (Zanette & Clinchy, 2019). The mere presence of predators or predator cues can induce changes in potential prey including increased stress, reduced time for mate finding, grooming, feeding, et cetera (Zanette & Clinchy, 2019). These individual level “nonconsumptive effects” (NCEs) can scale up, and cumulatively account for over half the impacts of predators on prey populations (Preisser & Bolnick, 2008). Recent efforts have been made to expand this concept to describe host–parasite interactions, sometimes under the label of “the ecology of disgust” (Buck et al., 2018; Daversa et al., 2021; Weinstein et al., 2018).

While parasite infection is known to reduce (i.e., suppress) host population size/growth (Hudson et al., 1998), NCEs of parasites are not thought to be a primary contributor to parasite‐mediated population suppression. One explanation is that the NCEs of parasites are smaller in magnitude than that of predators and anti‐parasite defenses are generally lower priority than anti‐predator defenses (Daversa et al., 2021; Horn et al., 2020; Koprivnikar & Penalva, 2015). Research into NCEs in host–parasite systems has typically focused on single‐host life stages, generally, the stage that is parasitized (Daversa et al., 2021; Koprivnikar & Penalva, 2015). For example, *Drosophila melanogaster* adults exposed to but not infected by *Gamasodes queenslandicus* mites had lower levels of lipids, glycogen, and protein (Benoit et al., 2020). However, recent research has found that ectoparasitic mites exert NCEs on multiple life stages of host flies (Horn, Robinson, et al., 2023). The apparent weakness of parasitic NCEs may result from artificially limiting our focus to specific life stages and not capturing the lifetime impact of NCEs.

In this study, we extend a previous effort to simulate host populations (only adult hosts) experiencing consumptive effects (CEs), that is infection, and NCEs of parasites by integrating new data on the NCEs of the facultatively parasitic mite *Macrocheles subbadius* on *Drosophila nigrospiracula* larvae (Horn, Robinson, et al., 2023). *Macrochelese subbadius* is a facultative ectoparasite that feeds on the hemolymph of adult flies which reduces the lifespan and reproductive output of host flies (Brophy & Luong, 2021; Polak, 1996). Flies defend themselves against infection through energetically demanding and time‐consuming defenses such as grooming, tarsal flicking, and short bursts of flight (Luong et al., 2015; Polak, 1996). These costly defenses likely drive NCEs through trade‐offs with other fitness‐related traits (Horn & Luong, 2021; Luong, Horn, & Brophy, 2017). Despite mites not infecting fly larvae, exposure to mites reduced the rate at which flies successfully reached adulthood (i.e. an NCE) (Horn, Robinson, et al., 2023). Horn, Robinson, et al. (2023) found that larvae avoided pupating in proximity to mites, potentially to avoid emerging in infectious environments, and this may come with feeding/developmental timing trade‐offs.

Computer simulations are a way to scale individual level effects to predict whether they will manifest population level changes (DeWitt et al., 2019). This is particularly useful, as experimental limitations (time, measurability, and controllability) have led to many more lab and individual‐organism studies than population level studies of NCEs (Sheriff et al., 2020). We extend our previously created matrix‐based framework (Horn et al., 2020) to simulate multi‐life stage populations with NCEs by incorporating the NCEs of parasite exposure during the larval stage (Horn, Robinson, et al., 2023). Our previous models found that NCEs did not significantly decrease the growth rates of simulated populations (Horn et al., 2020). However, elasticity analysis suggested the earlier mites affect flies in their lifespan the larger the impacts they have on host population growth (Horn et al., 2020). Therefore, we hypothesized NCEs experienced early in life, that is, as larvae, would have larger effects on population growth than infection or NCEs on adults. We, therefore, predicted the population size (# flies) of simulated fly populations would be lower in models with NCEs on larvae than in models with NCEs on just adults, or NCEs and infection of adults. We also model a mite‐free condition (no NCEs or CEs) and a “wild” condition (NCEs on all life stages + infection) to evaluate the relative importance of infection and NCEs on different life stages. This study is the first to test the importance of NCEs on different life stages for population growth effects. Determining if parasitic NCEs are capable of host population suppression would suggest a novel role of parasites in their ecological communities.

## 2 METHODS

Following the empirical findings of Horn, Robinson, et al. (2023) that the mere presence of an ectoparasite mite (*Macrocheles subbadius*), that is only known to infect adult stages of *Drosophila nigrospiracula*, impacted the survival of fly larva, we incorporated this data into an existing modeling framework to test the cumulative population‐level consequences of NCEs (Horn et al., 2020). The previous model fixed larval survival in order to investigate the NCE on adults, but in this new simulation, we allow larval survival to vary depending on the presence of parasites. Lifetime effects of mite exposure/infection are summarized in Table 1 along with data sources. In our models, we set daily survival to approximate these lifetime effects. Thus, when no parasites were present larval daily survival was set to 0.9716 and when parasites were present larval survival was set to 0.8965, derived from empirical survival in an experimental setting (Horn, Robinson, et al., 2023). Additionally, we tuned the daily probability of infection in adults to approximate the overall percentage of adults parasitized in mesocosm experiments (41%; Horn, Liang, & Luong, 2023).

**TABLE 1:** Summary of the consumptive and nonconsumptive effects (and 95% confidence intervals) of the mite *Macrocheles subbadius* on different life stages of the cactus fly *Drosophila nigrospiracula*.

Fly life stage	NCEs of mites	CEs of mites	Data source(s)
Eggs	No decrease in hatch rate among eggs laid near mites.	Treated as 0^a^	Mierzejewski et al. (2019)
Larvae/Pupae	Reduced survival by 61%, (16.3–28.3) to adulthood	Negligible infection observed	Horn, Robinson, et al. (2023)
Adults	Reduced lifespan 21%, (2.0–12.2) & reproductive output 13%, (−0.4–9.0)	Fecundity: 101.9% (32.2–175.0) Longevity 67.0% (9.6–19.8)	Horn and Luong (2018); Polak (1996)
Rates	Assumed all individuals experience NCE	41% (0.3164–0.5036) prevalence among adults	Horn, Liang, and Luong (2023)

*Note*: Rate of effects is also provided. Values are given as: Proportion/percent difference (95% confidence interval).

^a^ *Macrocheles* spp. feed on eggs; however, this is likely constant across conditions and the specific rate has not been measured in *M. subbadius*.

Data come from laboratory fly cultures founded from flies caught on wild cacti (Sonoran desert, AZ, USA), and experimental flies were from parasite‐naïve parents and grandparents (Horn & Luong, 2021). Data on the CE of infection on female survival and fecundity were collected from Polak, 1996. NCEs on adult flies were measured in Horn and Luong (2018). NCEs on pre‐adult flies are from Horn, Robinson, et al., 2023. In experiments that measured NCEs, flies (larval and adults) were separated from mites with a physical barrier (mesh) that prevented infection (Horn and Luong 2018; Horn, Robinson, et al., 2023). Other macrochelid species have been observed eating *Drosophila* eggs; however, data on oophagy do not exist for the *D. nigrospiracula—M. subbadius* association. The effect of egg consumption is presumably random and would not influence relative differences between the scenarios we model. Therefore, we did not incorporate egg consumption (Table 1).

With these data‐driven modifications in the modeling framework, we were able to simulate the population‐level effects of larval NCEs, adult NCEs, and/or CEs (infective costs) of parasitism on flies through six scenarios. For each scenario, we simulated 1000 populations of flies over 100 days starting from an initial dispersal of 50 adult flies to mimic the ephemeral habitat (cactus rot) these flies experience in the wild. In all cases, we plot the total adult population through time to visualize the population level consequences of parasitism. For a complete description of the framework used in the previous models see Horn et al. (2020); for code used in the models here see (doi: 10.17605/OSF.IO/NWAQS). Sensitivity analysis is available in Horn et al. (2020).

The scenarios were as follows. Scenario‐A is a baseline where no parasites are present, and the populations are unaffected by either the NCEs or CEs of parasites. In scenario‐B, only adults experience NCEs but not CEs, representing the fear‐only component of parasitism. In scenario‐C, larvae experience NCEs but no CEs, as the parasite is an ectoparasite of only the adults (Horn, Robinson, et al., 2023). In scenario‐D, both the larvae and adults experienced NCEs but there were no CEs. Scenario‐E represents the total effect of parasitism (NCE and CE) on adults only. Finally, scenario‐F represents the “wild” condition where both larva and adult experience NCEs and adults additionally suffer CEs.

A scenario that incorporates CEs without NCEs was not modeled. The data from previous sources were collected under semi‐natural conditions (i.e., flies are free to groom, move, were not anesthetized during infection, etc.). As a result, infected flies were also always exposed to mite cues, that is infected flies also experienced NCEs simultaneously. Thus, it was not possible to collect data that would permit modeling only CEs while maintaining semi‐natural conditions.

## 3 RESULTS

**FIGURE 1:**
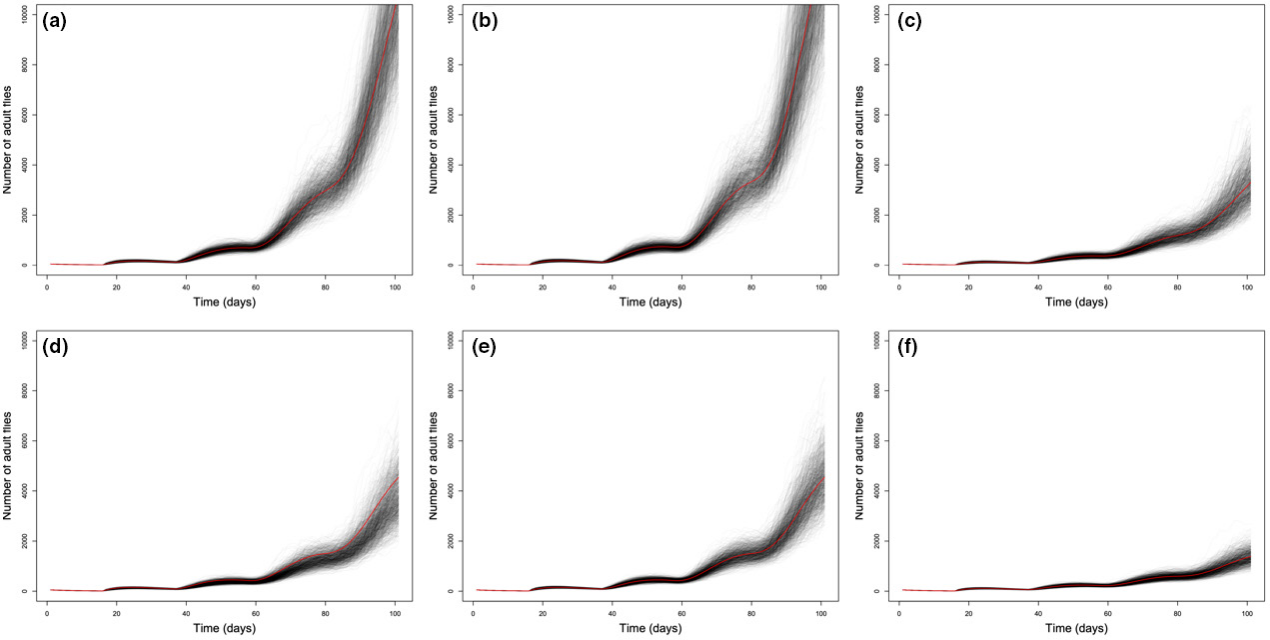
Simulations of 1000 populations of adult flies through 100 days starting with 50 dispersing flies, for scenarios with varying effects of parasites (combinations of consumptive effects, CEs, and nonconsumptive effects, NCEs). Each black line represents one simulated population; the red line is the average. We modeled six scenarios (a–f). Scenario A is the baseline scenario where flies are free from parasites (no NCEs or CEs). Scenario B is where only adults suffer the NCEs of parasitism. Scenario C is where only larvae suffer the NCEs of parasitism. Scenario D adults and larvae experience nonconsumptive effects but there is no infection. Scenario E adults suffer the total effects of parasitism, both NCEs and CEs, while larvae suffer no effects of parasitism. Scenario F represents “wild” conditions where adults suffer the total effects of parasitism (NCEs and CEs) and larvae suffer NCEs (larvae do not suffer CEs as the parasite is an ectoparasite of adults only).

We found that the NCEs on larva had the biggest impact on overall populations, reducing the population, on average, by 70.1% over the baseline (Scenario C vs. Scenario A in Figure 1). By contrast, adult NCEs alone (Scenario B) actually increased the population by 17.0% as a result of the over compensatory egg production (Horn et al., 2020). When NCEs on both adults and larvae were combined, without infection, (Scenario D) the population was reduced by 65.1%. By comparison, the combination of NCEs and CEs on adults (Scenario E) reduced the population by 58.6%. Thus, NCEs on larvae were comparable to the combined NCEs and CEs on adults. The total effect (NCE + NC) of parasitism on both the adults and larva, that is, “wild” conditions (Scenario F), reduced the population by 87.5%, on average (Figure 1). In scenarios involving adult infections (scenarios E and F), the overall percentage of adults parasitized was 41.7% and 39.3% respectively, approximately matching the 41% observed in Horn, Liang, and Luong (2023).

## 4 DISCUSSION

In recent years, the ecology of fear framework has been increasingly applied to parasite–host systems (Buck et al., 2018; Mierzejewski et al., 2019). As with predator–prey systems, evidence for population level effects of NCEs on hosts has been elusive (Horn et al., 2020; Sheriff et al., 2020). Here, we used simulations based on empirical observations of host behavior and survival in the lab to scale individual‐level effects to population‐level consequences that would otherwise be difficult to measure in the field. In simulations that mimic the natural setting where adults suffer both NCEs and CEs and the larvae only suffer NCE, we found that nonconsumptive effects during larval exposure accounted for approximately 80% of the observed population decline. Adults were able to compensate for NCEs on a population level by producing slightly more eggs per day (Horn et al., 2022), but this was insufficient to make up for the decline in survival due to parasite exposure during the larval stage in simulations including adult and juvenile NCEs. While previous work on NCEs in host–parasite systems has naturally focused on the life stage of the host that suffers parasitism, our empirical results (Horn, Robinson, et al., 2023) and simulations suggest that the fear of parasitism extends to other life stages, even if the threat is not imminent.

The current models do not incorporate mites feeding on fly eggs (Table 1). The rate at which *M. subbadius* feeds on *D. nigrospiracula* eggs is not known. However, the congeneric mite *Macrocheles muscaedomesticae* consumed on average 5.4 *Musca domesticae* (Diptera: Muscidae) eggs per day (Safaa et al., 2014). In this study, we were ultimately interested in the relative differences between the conditions modeled, and egg consumption is likely random and therefore unlikely to change the relative outcomes between conditions. Likewise, *M. muscaedomesticae* was also observed consuming 3.8 house fly larvae per day (Safaa et al., 2014). *M. muscaedomesticae* is substantially larger than *M. subbadius*, which has not been observed feeding on larvae, and as such may be better able to consume fly larvae. Although, the possibility remains that *M. subbadius* may have unidentified consumptive effects on fly populations. Regardless, data on larval and egg consumption may improve the predictive power of future models, but prediction of wild population dynamics was not the primary goal of this study. Further research is needed, including radiolabeling experiments (Polak, 1996), to conclusively determine the rate at which (if at all) *M. subbadius* feeds on fly larvae.

NCEs can potentially lead to intergenerational effects (MacLeod et al., 2022). Furthermore, over multiple generations, NCEs may shape the coevolution of natural enemies (Ydenberg et al., 2023; Zhang et al., 2022). We did not model intergenerational effects or coevolution as our model was based on empirical data of larvae from parasite‐naïve parents (Horn, Robinson, et al., 2023). However, work in predator–prey systems has shown cross‐generational effects (Sheriff et al., 2009). Juvenile hares from mothers exposed to predators (canine) before birth were lighter than juveniles from unexposed mothers (Sheriff et al., 2009). This effect was likely mediated by cortisol levels (Sheriff et al., 2009). These vertebrate–predator interactions may provide data for modeling cascading inter‐generational NCEs. A modeling approach across vertebrate and invertebrate systems may help address the lack of evidence for NCEs suppressing victim populations (Sheriff et al., 2020).

Our simulations concentrated on larval survival, however, there is empirical evidence suggesting *D. nigrospiracula* larvae exposed to *M. subbadius* had lower body mass upon reaching adulthood than adults that did not encounter mites (Horn, Robinson, et al., 2023). Such cascading inter‐lifestage NCEs have also been observed in fly‐spider interactions (Krams et al., 2016). *Drosophila melanogaster* larvae exposed to spider predators had altered body composition as adults—namely reduced size and reduced C:N ratios (Krams et al., 2016). At least in *D. nigrospiracula*, smaller body size correlates to lower female fecundity and reduced mating success among males (Polak, 1996). Inter‐lifestage effects may be a potentially fruitful avenue for further experimental and computational research. Additionally, there is evidence that NCEs of predation may influence pace‐of‐life characteristics such as developmental/reproductive timing (Montiglio et al., 2018). Our results may therefore underestimate the impacts of NCEs on host population growth. Future mechanistic models of population growth could incorporate NCEs on mass, developmental timing, and body composition on fecundity as well as inter‐generational effects (Benoit et al., 2020; Krams et al., 2016; Sheriff et al., 2009).

In our simulations, mites and adult flies arrive at a habitat simultaneously. This assumption is reasonable as *Macrocheles* have a rapid generation time (<1 week), mature in <48 hours, and are haplodiploid. One female mite, even unmated, can therefore generate a population by mating with their male offspring (Luong & Subasinghe, 2017). However, dispersal has been hypothesized to facilitate escaping parasites (Terui et al., 2017; Baines & Shaw, 2024). In this hypothesis, early colonizers receive a “head start” at new habitats before parasites are imported by late dispersers. This possibility is supported by the observation that infection intensity increases as cactus rots age, that is, late dispersers are more likely to be heavily infected as conditions deteriorate and mites are more inclined to parasitize flies (Johnston & Heed, 1976; Luong, Brophy, et al., 2017). Future models could incorporate a time lag between fly arrival and realized risk of infection, which increases as the rot ages. This may affect the relative importance of NCEs and CEs depending on the temporal dynamics of parasite pressure. However, the larval stages of insects cannot fly, and thus cannot minimize the NCE they experience through dispersal. Likewise, if there are NCEs that persist into adulthood following larval exposure, they are unlikely to be relieved by dispersal (Krams et al., 2016). Thus, inter‐lifestage NCEs may be of particular importance in understanding population dynamics among potential hosts and prey.

Research increasingly supports the idea that parasites have substantial ecological roles beyond infection per se (Dunne et al, 2013; Lafferty et al., 2006; Hudson et al., 2006; Macleod & Luong, 2024). Our results here suggest parasites may influence host population size through their NCEs, that is, without infection. NCEs on early life stages led to substantial decreases in population size; whereas, NCEs on adults only could even increase population size through compensatory reproduction (Figure 1b vs. Figure 1c). Future projects could use data on cascading inter‐generational and inter‐lifestage NCEs from vertebrate and invertebrate systems to make mechanistic models of NCE‐driven population suppression (Krams et al., 2016; Sheriff et al., 2009). Our research fits into a broader trend recognizing parasites are highly ecologically relevant beyond the effects of infection and suggests new avenues by which they may shape their communities.

## AUTHOR CONTRIBUTIONS

**Collin J. Horn:** Conceptualization (lead); data curation (lead); investigation (equal); writing – original draft (lead); writing – review and editing (lead). **Lien T. Luong:** Resources (equal); writing – review and editing (equal). **Darcy R. Visscher:** Conceptualization (equal); formal analysis (lead); investigation (equal); methodology (lead); software (lead); visualization (lead); writing – original draft (equal).

## CONFLICT OF INTEREST STATEMENT

No author has a conflict of interest to report.

## CODE ACCESSIBILITY

Code is available on OSF (DOI 10.17605/OSF.IO/NWAQS).

## Data Availability

This simulation study uses data from the literature (sources in Table 1). The original code is available at Open Science Foundation (DOI 10.17605/OSF.IO/NWAQS).
